# Health aspects, nutrition and physical characteristics in matched samples of institutionalized vegetarian and non-vegetarian elderly (> 65yrs)

**DOI:** 10.1186/1743-7075-8-37

**Published:** 2011-06-14

**Authors:** Peter Deriemaeker, Dirk Aerenhouts, Dolf De Ridder, Marcel Hebbelinck, Peter Clarys

**Affiliations:** 1 Department of Human Biometry and Biomechanics, Faculty of Physical Education and Physiotherapy, Vrije Universiteit Brussel, Pleinlaan 2, 1050 Brussels, Belgium

## Abstract

**Background:**

Epidemiological studies indicate that a well balanced vegetarian diet offers several health benefits including a lower prevalence of prosperity diseases in vegetarians compared to omnivores. It was the purpose of the present study to compare nutritional and physical characteristics in matched samples of institutionalized vegetarian (V) and non-vegetarian (NV) elderly.

**Methods:**

Twenty-two female and 7 male V (females: 84.1 ± 5.1yrs, males: 80.5 ± 7.5yrs) and 23 female and 7 male NV (females: 84.3 ± 5.0yrs, males: 80.6 ± 7.3yrs) participated. All subjects were over 65 years of age, and free of major disease or physical handicap. Dietary intake, blood profile, anthropometrics, and handgrip strength were determined.

**Results:**

Mean daily energy intake was 6.8 ± 2.0MJ in V females, and 8.0 ± 1.4MJ in the NV females, only the V did not reach the recommended value of 7.8 MJ. Male V and NV had a mean daily energy intake of 8.7 ± 1.6MJ and 8.7 ± 1.2MJ respectively (RDI: 8.8 MJ). Mean carbohydrate intake was significantly below the RDI in NV only (female V: 47.8 ± 7.5E%, female NV: 43.3 ± 4.6E%, male V: 48.1 ± 6.4E%, male NV: 42.3 ± 3.6E%), while protein (female V: 17.3 ± 3.4E%, female NV: 19.5 ± 3.5E%, male V: 17.8 ± 3.4E%, male NV: 21.0 ± 2.0E%), and saturated fat intake (female V: 25.4 ± 8.2 g/day, female NV: 32.2 ± 6.9 g/day, male V: 31.4 ± 12.9 g/day, male NV: 33.4 ± 4.7 g/day) were too high in both V and NV. Mean micronutrient intakes met the RDI's in all 4 groups. Mean blood concentrations for vitamin B12, folic acid, iron, and calcium were normal in all 4 groups. Mean zinc blood serum was below the reference value in all groups, whereas estimated zinc intake was in agreement with the RDI. The mean blood cholesterol concentration was above the 200 mg/dl upper limit in the V group (213 ± 40 mg/dl) and below that limit in the NV (188 ± 33 mg/dl) group. Mean BMI was 26.1 ± 4.7 kg/m^2 ^in the female V, 26.8 ± 3.7 kg/m^2 ^in the female NV, 23.5 ± 3.7 kg/m^2 ^in the male V, and 25.2 ± 4.2 kg/m^2 ^in the male NV. V and NV scored below the reference values for the handgrip strength test.

**Conclusions:**

Generally, our results show a similar profile for V and NV concerning dietary intake, blood values, and physical characteristics. Attention should be paid to the intake of mono- and disaccharides and saturated fats in the diet of both V and NV. This study indicates that a vegetarian lifestyle has no negative impact on the health status at older age.

## Background

Epidemiological studies on vegetarians show that appropriately planned vegetarian diets are healthy and nutritionally adequate [1-5]. Compared to omnivorous diets, vegetarian diets can provide several health benefits [6-9]. However, these positive health-related outcomes in vegetarians may be influenced by factors other than dietary practices. Regular physical activity and the avoidance of harmful practices such as smoking and excessive drinking, and being more "health conscious" in general are influencing factors [10]. Most studies compared a self-selected vegetarian group with standard population references [1]. In order to truly attribute the health advantages associated with a vegetarian lifestyle, it is necessary to compare the vegetarian subjects with an adequate reference sample [11-14]. In such a design the vegetarians are compared with a comparable non-vegetarian sample to exclude possible confounding factors.

The national nutritional surveys in Belgium and the Netherlands revealed a poor eating pattern and lack of physical activity in the Belgian and Dutch population [15-17]. The reported macronutrient distribution in the elderly (65-75yrs) (17% protein, 39% fat, 44% carbohydrate) was similar in both countries and not in accordance with the reference values proposed by their respective Health Councils [17,18]. The surveys indicated a too high intake of saturated fat and protein with meat, fish, eggs and dairy products as the main protein sources, a negligible consumption of plant-based products (seeds, nuts, pulses, tofu, quorn and tempeh), and an intake of fruit and vegetables far below the recommendations [15-17].

In general, energy intake decreases throughout adulthood, leading to a reduced macro- and micronutrient intake at older age [19,20]. Knowledge of the nutritional status of elderly people is of particular importance, since the elderly population has a higher prevalence of chronic medical conditions [21,22], malnutrition [23], and at the same time an increased prevalence of many chronic diseases which are associated with nutritional status [24-26]. Nonetheless, there are few studies on the nutritional status of elderly vegetarians, and dietary studies on vegetarians usually include only a few elderly, if any [1-5]. Although the dietary intake in vegetarians appears favorable with respect to chronic disease risk factor profile, deficiencies in certain nutrients have been found [27-32]. Of these latter studies very few have been carried out in elderly subjects [32]. Although the number of vegetarians is relatively small in the Belgian and Dutch population [15], it is important to study their nutritional status, as in certain day care centers and senior citizens homes vegetarian meals are served. Since there is a growing interest in healthy diets and vegetarian nutrition, particularly in the elderly [33-35], a matched samples study on institutionalized elderly vegetarians compared with institutionalized non-vegetarians may contribute to the present knowledge of the dietary pattern in the elderly.

It is the purpose of the present study to compare the nutritional, health and physical status in comparable groups of vegetarian (V) and non-vegetarian (NV) elderly in order to gain more insight into the adequacy of a vegetarian diet at older age in particular.

## Methods

### Participants

Twenty-two female and 7 male V elderly living in a vegetarian senior citizens home in the Netherlands (female: 84.1 ± 5.1yrs, male: 80.5 ± 7.5yrs) and 23 female and 7 male NV elderly living in a regular senior citizens home in the Dutch speaking part of Belgium (female: 84.3 ± 5.0yrs, male: 80.6 ± 7.3yrs) volunteered to participate in this study. All subjects were "apparently healthy", which was defined as free of major disease or physical handicap. In agreement with the university ethics committee, all participants received explanation about the purpose and procedures of the study and signed an informed consent statement before participating.

### Measures and procedure

Dietary intake (Food Frequency Questionnaire, FFQ), blood profile, anthropometrics, and handgrip strength were registered.

During our visit at their respective senior citizens homes, all V and NV participants completed a validated semi-quantitative 104 items FFQ [36] to estimate their dietary intakes over the last 6 months. A standard portion size and 9 possible food-frequency categories, ranging from never or less than 1 time per month to 6 or more times per day, were given for each food items. The FFQ was completed in the presence of the researcher, allowing clarification and help when necessary.

Blood samples were collected after an overnight fast and analysed for haemoglobin, red cell count, serum iron, transferrine, ferritine, white cells, ureum, albumin, serum calcium, serum zinc, vitamin B12, folic acid, triglycerides, total cholesterol, HDL-cholesterol and LDL-cholesterol in the clinical biology laboratory of the university hospital of the Vrije Universiteit Brussel, following validated standard procedures.

Anthropometric variables were registered in order to determine the body mass index (BMI, kg/m^2^) and the waist-hip ratio. Additionally, triceps and subscapular skinfolds and upperarm girth were taken. The handgrip strength was measured using a hand dynamometer grip strength meter. Measurements were carried out according to standardized techniques [37,38].

### Statistical analysis

In case of different reference values [17,18] for men and women, males and females were analysed separately. After testing for normality (Kolmogorov Smirnov Goodness of Fit test) the data at scale level were compared using an independent sample t-test for comparisons between the female V and NV. Because of the small number of male subjects, the Mann-Whitney test was used for the comparisons between the male V and NV groups. One sample t-tests were used for comparisons with the recommended daily intakes (RDI). Statistical analyses were performed using SPSS, and the significance level was set at 0.05.

## Results

Table 1 shows that the mean daily energy intake of 6.8 ± 2.0MJ in V females was not according the RDI of 7.8MJ, in contrast to the 8.0 ± 1.4MJ in the NV females. Male V and NV met the RDI of 8.8MJ with a mean daily energy intake of 8.7 ± 1.6MJ and 8.7 ± 1.2MJ respectively. Mean protein intake (female V: 17.3 ± 3.4E%, female NV: 19.5 ± 3.5E%, male V: 17.8 ± 3.4E%, male NV: 21.0 ± 2.0E%) was too high in the female V and female and male NV. The female V had a lower protein intake compared to the female NV. Mean carbohydrate intake was significantly lower than the RDI in the NV only (female V: 47.8 ± 7.5E%, female NV: 43.3 ± 4.6E%, male V: 48.1 ± 6.4E%, male NV: 42.3 ± 3.6E%). The female V had a higher carbohydrate intake compared to their NV counterparts. Mono- and disaccharide intake was too high in female NV and male V. Polysaccharide intake was too low in all 4 groups compared to the RDI. Dietary fibre intake was sufficient in all 4 groups. Total fat intake (female V: 34.7 ± 7.2E%, female NV: 35.2 ± 4.5E%, male V: 32.8 ± 7.9E%, male NV: 32.6 ± 3.0E%) was in accordance with the RDI in all 4 groups. Saturated fat intake was significantly above the maximum RDI (females: 21 g/day, males: 23 g/day) for both V and NV females and male NV. Comparable results in the V and the NV samples were found for the mono- and poly-unsaturated fatty acid intake. Alcohol intake was significantly lower in the female V compared to the NV, while no difference was found between the male V and NV. Mean daily water intake was sufficient in all groups. Mean vitamin and mineral intakes met the RDI in both groups (Table 2).

**Table 1:** Daily energy, macronutrient and water intake in vegetarian (V) and non-vegetarian (NV) elderly males (m) and females (f) (mean ± SD)

Variable	Gender	V	NV	RDI
Energy (MJ)	m	8.7 ± 1.6	8.7 ± 1.2	8.8
	f	6.8 ± 2.0*,†	8.0 ± 1.4	7.8
Protein (E%)	m	17.8 ± 3.4	21.0 ± 2.0†	10-15
	f	17.3 ± 3.4*,†	19.5 ± 3.5†	10-15
Total carbohydrate (E%)	m	48.1 ± 6.4	42.3 ± 3.6†	50-55
	f	47.8 ± 7.5*	43.3 ± 4.6†	50-55
Mono- and disaccharides (g)	m	170 ± 37†	137 ± 35	131
	f	137 ± 50	133 ± 30†	117
Polysaccharides (g)	m	66 ± 22†	56 ± 3†	158
	f	50 ± 24†	59 ± 14†	140
Fibre (g)	m	28 ± 9	36 ± 10	27
	f	28 ± 18	32 ± 11	27
Total fat (E%)	m	32.8 ± 7.9	32.6 ± 3.0	30-35
	f	34.7 ± 7.2	35.2 ± 4.5	30-35
Saturated fat (g)	m	31.4 ± 12.9	33.4 ± 4.7†	23
	f	25.4 ± 8.2*,†	32.2 ± 6.9†	21
MUFA (g)	m	28.8 ± 11.7	39.1 ± 4.2†	28
	f	22.8 ± 10.1*	37.1 ± 6.1†	25
PUFA (g)	m	14.3 ± 2.8†	13.8 ± 1.3†	28
	f	13.4 ± 7.0†	12.2 ± 3.4†	25
Cholesterol (mg)	m	181 ± 56	208 ± 21	< 300
	f	161 ± 69	205 ± 46	< 300
Alcohol (E%)	m	1.7 ± 3.1	13.2 ± 13.1	< 4
	f	0.3 ± 0.9*	5.6 ± 7.7	< 4
Water (ml)	m	2050 ± 277	2224 ± 214	1600-2400
	f	1663 ± 452*	2166 ± 538	1600-2400

*Significantly different with NV (p < 0.05)

†Significantly different from Recommended Daily Intake (RDI) (p < 0.05)

MUFA: mono-unsaturated fatty acids

PUFA: poly-unsaturated fatty acids

**Table 2:** Daily micronutrient intake in vegetarian (V) and non-vegetarian (NV) elderly males (m) and females (f) (mean ± SD)

Micronutrients	Gender	V	NV	RDI
Calcium (mg)	m	1096 ± 371	969 ± 399	800-1000
	f	894 ± 306	858 ± 265	800-1000
Iron (mg)	m	15.7 ± 3.4†	13.2 ± 1.5†	9
	f	12.5 ± 5.6†	12.8 ± 3.0†	8
Zinc (mg)	m	13.3 ± 3.8	11.9 ± 1.8†	10
	f	13.0 ± 8.3	12.0 ± 4.3†	9
Sodium (mg)	m	4083 ± 1231	4911 ± 831†	575-3500
	f	2861 ± 907*	4944 ± 971†	575-3500
Potassium (mg)	m	3976 ± 794*,†	3127 ± 395	1600-3100
	f	3217 ± 963	3203 ± 580	1600-3100
Phosphorus (mg)	m	1672 ± 429†	1395 ± 443	1000
	f	1396 ± 658†	1322 ± 307†	1000
Magnesium (mg)	m	388 ± 129*	248 ± 46†	325
	f	340 ± 190*	252 ± 74	275
Vit A (mg)	m	0.70 ± 0.21*	1.13 ± 0.10†	0.7
	f	0.55 ± 0.21*	1.06 ± 0.21†	0.6
Beta-carotene (mg)	m	1.30 ± 0.92	1.07 ± 0.49	-
	f	1.01 ± 0.41	1.04 ± 0.42	-
Vit B1 (mg)	m	1.60 ± 0.61*,†	0.99 ± 0.16	1
	f	1.41 ± 1.22	1.08 ± 0.45	1
Vit B2 (mg)	m	1.82 ± 0.41*	1.20 ± 0.20†	1.5
	f	1.51 ± 0.62	1.24 ± 0.36	1.3
Vit B6 (mg)	m	1.50 ± 0.42†	1.56 ± 0.18†	1.1
	f	1.21 ± 0.42*,†	1.47 ± 0.26†	1.0
Vit D (μg)	m	0.31 ± 0.01	0.27 ± 0.05	-
	f	0.11 ± 0.02*	0.23 ± 0.08	-
Vit C (mg)	m	162 ± 47*,†	96 ± 35	70
	f	149 ± 82†	123 ± 46†	70

*Significantly different with NV (p < 0.05)

†Significantly different from Recommended Daily Intake (RDI) (p < 0.05)

Blood concentrations for vitamin B12, folic acid, iron, and calcium were within the recommended values in both groups (Table 3). Zinc blood serum was below the reference value in both groups. The mean blood cholesterol concentration was above the 200 mg/dl upper limit in the V group (213 ± 40 mg/dl) and below that limit in the NV (188 ± 33 mg/dl) group. As to the blood biochemistry, a comparable number of subjects in both groups did not reach the reference values.

**Table 3:** Blood profile in vegetarian (V) and non-vegetarian (NV) elderly males (m) and females (f) (mean ± SD)

Parameter	Gender	V (n, n a-typ.)^(a)^	NV (n, n a-typ.)^(a)^	**Ref**.
Haemoglobin (mmol/l)	m	13.4 ± 0.9 (4, 2)	13.6 ± 1.2 (6, 2)	13-16.5
	f	12.8 ± 1.1 (18, 3)	13.1 ± 1.6 (20, 3)	11.8-14.5
Red cell count (10^6^/mm^3^)	m	4.6 ± 0.3 (4, 0)	4.5 ± 0.5 (6, 0)	4.2-5.7
	f	4.3 ± 0.5 (21, 2)	4.4 ± 0.5 (20, 2)	3.9-5.0
Serum iron (mg/dl)	m+f	76 ± 25 (24, 4)	82 ± 36 (26, 1)	50-170
Transferrine (mg/dl)	m+f	243 ± 33* (25, 1)	217 ± 37 (26, 1)	181-332
Ferritine (mg/l)	m	47 ± 26 (4, 1)	161 ± 165 (6, 0)	30-400
	f	70 ± 46 (21, 1)	158 ± 180 (20, 1)	13-150
White cells (10^3^/mm^3^)	m+f	6.4 ± 1.5* (22, 0)	7.8 ± 2.5 (26, 5)	3.6-9.6
Ureum (mg/dl)	m+f	36.9 ± 11.0 (24, 8)	42.5 ± 23.5 (26, 14)	15-40
Albumin (g/dl)	m+f	3.9 ± 0.3* (24, 3)	3.7 ± 0.3 (26, 3)	3.7-5.0
Serum calcium (10^3^/mm^3^)	m+f	8.8 ± 0.4 (24, 5^(b)^)	9.0 ± 0.4 (26, 1)	8.6-9.8
Serum zinc (mg/dl)	m+f	62.0 ± 11.1† (24, 20)	64.5 ± 12.2† (26, 18)	70-150
Total cholesterol (mg/dl)	m+f	213 ± 40*,† (24, 18)	188 ± 33 (25, 12)	< 200
LDL (mg/dl)	m+f	121 ± 30 (24, 15)	111 ± 26 (24, 11)	< 115
HDL (mg/dl)	m+f	54 ± 15 (24, 4)	51 ± 12 (26, 2)	> 40
Triglycerides (mg/dl)	m+f	180 ± 106 (24, 10)	149 ± 103 (26, 6)	< 180
Vitamin B12 (mg/l)	m+f	0.58 ± 0.60 (22, 7)	0.46 ± 0.46 (26, 7)	0.22-0.94
Folic acid (mg/l)	m+f	9.8 ± 5.7 (22, 6)	7.3 ± 6.0 (26, 6)	2.0-14.0

^(a)^(n: number of subjects studied, n a-typ.: number of subjects with a-typical values)

^(b)^The values were close to the cut-off point, i.e. 8.1, 8.3, 8.4, 8.4, 8.4

*Significantly different with NV (p < 0.05)

†Significantly different from reference values (Ref.) (p < 0.05)

Mean BMI did not differ between V and NV with respective BMI values of 26.1 ± 4.7 kg/m^2 ^in the female V,26.8 ± 3.7 kg/m^2 ^in the female NV, 23.5 ± 3.7 kg/m^2 ^in the male V and 25.2 ± 4.2 kg/m^2 ^in the male NV (Table 4). The waist-hip ratio was comparable between V and NV groups, and was below the upper reference value of 0.99 for males and 0.90 for females [39]. As to the strength test both groups scored below the reference values of 40 kg for males and 25 kg for females [40]. There were no differences between the V and NV groups.

**Table 4:** Anthropometry and strength of vegetarian (V) and non-vegetarian (NV) elderly males (m) and females (f) (mean ± SD)

Variable	Gender	V	NV	**Ref**.
Body height (cm)	m	174.6 ± 7.6	169.0 ± 7.0	
	f	154.5 ± 8.1*	158.8 ± 4.1	
Body weight (kg)	m	72.2 ± 14.9	68.7 ± 8.7	
	f	62.2 ± 10.8	67.6 ± 10.2	
BMI (kg/m^2^)	m	23.5 ± 3.7	25.2 ± 4.2	19-25
	f	26.1 ± 4.7	26.8 ± 3.7	19-25
Waist-Hip ratio	m	0.91 ± 0.10	0.94 ± 0.04	0.99
	f	0.90 ± 0.10	0.90 ± 0.06	0.90
Triceps skinfold (mm)	m	10.6 ± 4.3†	13.0 ± 2.8	15.0
	f	12.7 ± 3.5†	11.2 ± 4.0†	21.6
Subscapular skinfold (mm)	m	12.2 ± 3.0†	11.9 ± 6.0	16.2
	f	9.2 ± 2.6*,†	13.5 ± 3.5†	19.0
Upperarm girth (cm)	m	27.9 ± 4.0	27.4 ± 3.0	28.3
	f	27.7 ± 2.9†	29.0 ± 3.9	30.3
Handgrip (kg)	m	29.0 ± 9.1†	24.7 ± 7.2†	40.3 ± 7.9
	f	17.5 ± 5.5†	16.1 ± 6.0†	25.4 ± 5.4

*Significantly different with NV (p < 0.05)

†Significantly different from reference values (Ref.) (p < 0.05)

## Discussion

A major problem is the difficulty to compare with reference values as no references for very old people (> 75yrs) exist. Therefore this study compares two matched samples of elderly citizen home people with a mean age of 84.1 ± 5.1yrs in female V, 84.3 ± 5.0yrs in female NV, 80.5 ± 7.5yrs in male V, and 80.6 ± 7.3yrs male NV, which is far above the cut-offs of most reference values.

The macronutrient intake was in agreement with the Belgian and Dutch food consumption surveys [17,18]. Only in male V, protein intake was within the recommendations, while for the other groups protein intake was too high. Mean carbohydrate intake was too low in the male and female NV compared to the RDI. Total fat intake met the RDI in all groups. The saturated fat intake was comparable and, except in the male V, significantly above the RDI in all groups. Mean daily energy intake was lower in V females compared to their NV counterparts and did not reach the recommended value of 7.8 MJ. Most studies found no differences in energy intake between V and NV [1-5]. The difference found between the female groups in this study can be due to several reasons. Female V had a lower protein and saturated fat intake and a higher carbohydrate intake compared to the female NV. The macronutrient profile in V females was closer to the recommendations compared to the NV females. However both groups had protein, saturated fat and carbohydrate intakes not in accordance with the RDI. Although only significant in the females, the V had a lower alcohol intake compared to the NV. The better macronutrient profile and a lower alcohol consumption may be attributed to the often cited "health consciousness" in vegetarians [1,10]. Mean vitamin and mineral intakes met the RDI in both groups, which indicates a varied choice of micronutrient dense foods in both groups. A study of Brants et al. [24] indicated a favourable food choice of independently living elderly vegetarians preparing their own meals compared to institutionalized elderly vegetarians. Compared to the institutionalized subjects studied by Brants et al. [24] our subjects (V and NV) show similar dietary shortcomings. Although the fact that vitamin B12, folic acid, iron, and calcium are often described as critical in a vegetarian diet, the mean blood concentrations of these parameters were normal in both groups. The main explanation can be found in the fact that our subjects were lacto-ovo vegetarians. While estimated zinc intake was sufficient, zinc blood serum was below the reference value in both groups. This indicates that the RDI may be underestimated for elderly as a consequence of their poor absorption due to aging. The mean blood cholesterol concentration was below the 200 mg/dl upper limit in the NV group and above that limit in the V group. These higher blood cholesterol levels are comparable with those found in elderly in Flanders where 86% of the males and 91% of the females between 65 and 69 yrs have blood cholesterol levels above 200 mg/dl [41]. Since the subjects in this study have a mean age over 80 years the values in the vegetarian subjects can be considered as normal. Moreover, several studies suggest that slightly higher cholesterol levels may even protect against infections and atherosclerosis [42-44]. According to these authors, values from 200 to 239 mg/dl are more appropriate in elderly (> 65 yrs) [42-44]. Comparable numbers of subjects not reaching blood reference values were found in both groups. Of note is that vitamin B12 deficiency is not always unique to vegetarians, although it is generally more difficult for vegetarians (especially vegans) to meet vitamin B12 requirements than it is for omnivores. The present results show a mean vitamin B12 status according to the reference values while a comparable number of subjects with atypical values were detected for the V and NV. Therefore a low vitamin B12 status is not likely to be related to neither the vegetarian nor the omnivorous diet. The present results confirm the advice of Elmadfa and Singer [45] that a regular monitoring of the vitamin B12 status in order to facilitate early detection of low vitamin B12 status and timely treatment before clinical manifestations can develop is required. These concerns should also be taken into account for the other blood parameters.

Mean BMI was around 25 and comparable between V and NV in both genders. This is in contrast with previous studies which showed vegetarians to have a lower BMI [7,46,47]. Appleby *et al*. [46] found a significant inverse association between dietary fibre and BMI, but this was not confirmed by our results. The lower reported saturated fat intake in female V as compared to the NV did not result in a significantly lower BMI or waist-hip ratio in female V. Except lower subscapular skinfolds in V females no anthropometric differences were found between the V and NV. This can be a consequence of working with small matched samples. The findings concerning the anthropometric characteristics indicate an adequate energy balance, also in the female V who reported an energy intake below the reference value. The waist-hip ratio was comparable between V and NV groups, and was below the upper reference value [39]. Price et al. [39] and Seidell [48] showed that for persons aged > 75 yrs, waist-hip ratio should be used instead of BMI because of the positive relationship between mortality and abdominal adiposity. Therefore we can consider that the elderly V and NV in the present study are not at increased risk. Regarding the handgrip strength both groups scored below the reference value and no differences were found between the V and NV. The anthropometric and physical differences compared to the reference values (65 - 75 yrs) may be due to the higher mean age (> 80 yrs) of the studied V and NV groups.

Since in this study V and NV did not substantially differ with respect to dietary intake and nutritional status, one should be careful with the conclusion that only dietary factors of a vegetarian diet cause different morbidity and mortality risks among V and NV.

A limitation of the present study may be the fact that the compared senior citizens homes in this study are located in two different regions (Belgium and the Netherlands). This is mainly due to the fact that, to our knowledge, there is but one exclusively vegetarian senior citizens home in the Netherlands and none in the Dutch speaking region of Belgium. Nonetheless, the national food consumption surveys in both regions showed similar shortcomings typical for European diets regarding the macro- and micronutrient intake. Another drawback is the non-randomized sampling of the two populations studied. More extensive studies have to be undertaken in order to gain more knowledge about the value of vegetarian diets in elderly people.

## Conclusion

The macronutrient profile in V females was closer to the recommendations compared to the NV females. However, both groups had macronutrient intakes not in accordance with the RDI. Blood values, anthropometrics and handgrip strength were comparable for V and NV. These diet-related health risk factors found in both V and NV can be corrected by additional nutritional interventions. Such interventions should be encouraged and monitored by the responsible authorities and caretakers of the senior citizens homes and institutions. The present results suggest that the shortcomings of health related characteristics and dietary intake are not likely to be specifically related neither to the vegetarian nor to the omnivorous diet. Finally, this study corroborates the assertion of many professional instances that a balanced, and well planned vegetarian diet can be a responsible choice also at older age [1].

## Competing interests

The authors declare that they have no competing interests.

## Authors' contributions

MH, DDR and PC conceived the study. PC and MH were responsible for the data collection. PD participated in its design and coordination, did the statistical analyses and wrote the manuscript. DA, MH, DDR and PC critically reviewed the manuscript for writing and intellectual content. All authors read and approved the final manuscript.
